# Genetic Testing for Global Developmental Delay in Early Childhood

**DOI:** 10.1001/jamanetworkopen.2024.15084

**Published:** 2024-06-05

**Authors:** Jiamei Zhang, Yiran Xu, Yun Liu, Ling Yue, Hongfang Jin, Yongqian Chen, Dong Wang, Mingmei Wang, Gongxun Chen, Lei Yang, Guangyu Zhang, Xiaoli Zhang, Sansong Li, Huiling Zhao, Yunxia Zhao, Guohui Niu, Yongqiang Gao, Zhijun Cai, Fan Yang, Changlian Zhu, Dengna Zhu

**Affiliations:** 1 Department of Rehabilitation Medicine, Third Affiliated Hospital of Zhengzhou University, Zhengzhou, China; 2 Henan Key Laboratory of Child Brain Injury and Henan Pediatric Clinical Research Center, Institute of Neuroscience and Third Affiliated Hospital of Zhengzhou University, Zhengzhou, China; 3 Kunming Children’s Hospital, Kunming, China; 4 Department of Neurological Rehabilitation, Children’s Hospital of Hebei Province, Shijiazhuang, China; 5 Qinghai Provincial Women and Children’s Hospital, Xining, China; 6 Lanzhou University Second Hospital, Lanzhou, China; 7 Department of Pediatric Neurology, Xi’an Children’s Hospital, Xi’an, China; 8 Department of Pediatric Neurology, Third Affiliated Hospital of Zhengzhou University, Zhengzhou, China; 9 Cipher Gene LLC, Beijing, China; 10 Center for Brain Repair and Rehabilitation, Institute of Neuroscience and Physiology, University of Gothenburg, Gothenburg, Sweden

## Abstract

**Importance:**

Global developmental delay (GDD) is characterized by a complex etiology, diverse phenotypes, and high individual heterogeneity, presenting challenges for early clinical etiologic diagnosis. Cognitive impairment is the core symptom, and despite the pivotal role of genetic factors in GDD development, the understanding of them remains limited.

**Objectives:**

To assess the utility of genetic detection in patients with GDD and to examine the potential molecular pathogenesis of GDD to identify targets for early intervention.

**Design, Setting, and Participants:**

This multicenter, prospective cohort study enrolled patients aged 12 to 60 months with GDD from 6 centers in China from July 4, 2020, to August 31, 2023. Participants underwent trio whole exome sequencing (trio-WES) coupled with copy number variation sequencing (CNV-seq). Bioinformatics analysis was used to unravel pathogenesis and identify therapeutic targets.

**Main Outcomes and Measures:**

The main outcomes of this study involved enhancing the rate of positive genetic diagnosis for GDD, broadening the scope of genetic testing indications, and investigating the underlying pathogenesis. The classification of children into levels of cognitive impairment was based on the developmental quotient assessed using the Gesell scale.

**Results:**

The study encompassed 434 patients with GDD (262 [60%] male; mean [SD] age, 25.75 [13.24] months) with diverse degrees of cognitive impairment: mild (98 [23%]), moderate (141 [32%]), severe (122 [28%]), and profound (73 [17%]). The combined use of trio-WES and CNV-seq resulted in a 61% positive detection rate. Craniofacial abnormalities (odds ratio [OR], 2.27; 95% CI, 1.45-3.56), moderate or severe cognitive impairment (OR, 1.69; 95% CI, 1.05-2.70), and age between 12 and 24 months (OR, 1.57; 95% CI, 1.05-2.35) were associated with a higher risk of carrying genetic variants. Additionally, bioinformatics analysis suggested that genetic variants may induce alterations in brain development and function, which may give rise to cognitive impairment. Moreover, an association was found between the dopaminergic pathway and cognitive impairment.

**Conclusions and Relevance:**

In this cohort study of patients with GDD, combining trio-WES with CNV-seq was a demonstrable, instrumental strategy for advancing the diagnosis of GDD. The close association among genetic variations, brain development, and clinical phenotypes contributed valuable insights into the pathogenesis of GDD. Notably, the dopaminergic pathway emerged as a promising focal point for potential targets in future precision medical interventions for GDD.

## Key Points

**Question:**

What are the implications of genetic testing for global developmental delay (GDD) in early childhood?

**Findings:**

In this cohort study of 434 children with GDD, a diagnostic positivity rate of 61% was identified when using trio whole exome sequencing combined with copy number variation sequencing. A thorough analysis expanded the scope of indications for genetic testing, and the pathogenesis of GDD was further elucidated using a bioinformatics approach.

**Meaning:**

These findings suggest that early use of combined genetic testing for GDD may diminish the misdiagnosis rate, elucidate the etiologic diagnosis, and lay the groundwork for identifying novel early diagnostic biomarkers and intervention targets.

## Teaser

This cohort study evaluates the utility of genetic testing for global developmental delay in young children in China and assesses potential targets for early intervention.

## Introduction

Global developmental delay (GDD) is a prevalent neurodevelopmental disorder marked by cognitive impairment. Most children with GDD develop intellectual disability (ID) after the age of 5 years, impacting their quality of life, physical abilities, and social functioning. With an overall prevalence rate of approximately 1%,^1,2,3^ ID ranks among the top 10 major global diseases.^4^ Hence, early and accurate etiologic diagnosis, coupled with targeted treatment, becomes particularly crucial for enhancing children’s health and alleviating the national health care burden.^5,6^

Genetic factors play a significant role in the pathogenesis of GDD.^7,8,9,10,11^ Currently, definitive biomarkers^12^ and precise treatment measures^13^ for GDD are lacking. Consequently, appropriate genetic testing and comprehensive evaluations are increasingly acknowledged as essential for comprehending the etiology of GDD and identifying innovative intervention strategies. These measures are vital for effectively managing GDD and offering hope for improved neurodevelopmental outcomes.

The predominant genetic causes in GDD and ID are linked to single nucleotide variations (SNVs), insertions/deletions (indels), and copy number variations (CNVs). Advances in next-generation sequencing technologies have demonstrated their ability to detect genetic material throughout the entire genome, thereby reducing diagnostic errors and increasing diagnostic rates for SNVs, indels, and CNVs in patients with GDD or ID.^14^ However, various genetic testing approaches still exhibit variations in the detection rate of genetic causes for GDD and ID.^15,16^ Compared with individual WES, trio-WES shows a 12%^15,17^ increase in diagnostic rates due to its enhanced identification of de novo variants and structural rearrangements within coding regions of DNA. Additionally, CNV-seq surpasses the limitations of conventional chromosomal microarray in detecting CNVs across the entire genome and identifying novel CNVs,^14,17,18^ making a sensitive and specific detection method.^19^ Despite these advancements, there have been relatively few reports on combined trio-WES and CNV-seq in early childhood with GDD.

The origin of GDD and ID remains unclear, with several geneticists directing their focus on synaptic development^20,21^ to elucidate disease models. A prevailing theory suggests that alterations in the functional states of nerve cells, potentially influenced by environmental factors, may lead to abnormal synaptic development.^22,23^ However, these insights are primarily derived from studies on specific disorders, such as Down syndrome, Fragile X syndrome, and Rett syndrome,^20,21,22,23^ and their universal applicability to all cases remains uncertain. Cognitive impairment, a prevalent symptom of GDD and ID, significantly influences the prognosis of these conditions. The development of cognitive functions is therefore a pivotal aspect in the onset and progression of these disorders. Accordingly, a focus on the advancement of cognitive function might offer a new strategy to unravel the pathogenesis of GDD.

Current genetic detection research predominantly focuses on single methods, particularly in the context of ID, and lacks investigations into the clinical applicability of early childhood GDD or the combined use of different genetic detection methods. Additionally, the study of etiology often concentrates solely on the pathogenesis of individual genes, providing an incomplete understanding of how genetic variations contribute to clinical manifestations. Recognizing these gaps, this research aimed to evaluate the utility of combining trio-WES with CNV-seq in patients with GDD and explore the potential molecular pathogenesis of GDD along with targets for early intervention.

## Methods

### Cohort Recruitment

This prospective, multicenter cohort study centered on children diagnosed with unexplained GDD who were either outpatients or inpatients in China from July 4, 2020, to August 31, 2023. We implemented the following inclusion criteria: (1) meeting the diagnostic criteria for GDD, as defined in the *Diagnostic and Statistical Manual of Mental Disorders* (Fifth Edition); (2) Gesell scale indicating a developmental quotient below 75 in at least 2 performance areas, including the adaptive performance area, which is related to cognitive development^24^; (3) children aged 12 to 60 months; and (4) securing a written informed consent for sequencing and analysis by the legal guardians. We excluded from this study children with GDD due to conditions such as cerebral palsy and spinal cord injuries leading to motor impairment as well as those with postnatal encephalitis, brain trauma, or cerebral hemorrhage resulting in acquired nervous system damage. Ethical approval for this study was obtained from the Ethics Committee of the Third Affiliated Hospital of Zhengzhou University. This report follows the Strengthening the Reporting of Observational Studies in Epidemiology (STROBE) reporting guideline for cohort studies.

### Analysis and Data Visualization Methods in MRI

Brain magnetic resonance imaging (MRI) was conducted for all enrolled patients. We present a detailed description of the techniques for analyzing and visualizing MRI data in the eMethods in Supplement 1.

### Trio-WES and CNV-Seq

Genomic DNA was extracted from blood samples collected from patients and their parents. The sequencing and data analysis process is detailed in the eMethods and eFigures 1 and 2 in Supplement 1).

### Bioinformatics Analysis

Gene Ontology (GO) enrichment analyses, encompassing biological process, cellular component, and molecular function, were conducted on the detected genes in the GDD cohort using R software, version 4.2.1 (R Foundation for Statistical Software). The list of genes from the GDD cohort underwent Kyoto Encyclopedia of Genes and Genomes (KEGG) pathway enrichment analysis through Metascape (eMethods in Supplement 1).^25^ Additionally, a similar method was used to analyze genes associated with cognition. The gene list from the GDD cohort was input into STRING,^26^ and pathways with a confidence level exceeding 40% were selected. Subsequently, the data were exported and imported into the Cytoscape.^27^ The cytoHubba plug-in was applied to identify key genes, and the results were visualized and analyzed. A competitive enzyme-linked immunosorbent assay was used for the detection of dopamine (eMethods in Supplement 1).

### Statistical Analysis

The case data were inputted into a computer, and a data table was generated using Microsoft Excel 2021 (Microsoft Corp). Categorical variable data were expressed as incidences or composition ratios, and group comparisons were performed using the χ^2^ test or Fisher exact test, with multigroup comparisons adjusted for false discovery rate correction. Continuous variable data were presented as medians and IQRs. Intergroup comparisons were executed using the Mann-Whitney *U* test, and multigroup comparisons were performed using the Kruskal-Wallis test, with false discovery rate correction. A binary logistic regression model was used to analyze the association between the percentage of patients with positive genetic expressions and clinical phenotypes. Statistical analyses were conducted using SPSS software, version 26.0 (SPSS Inc) and R, version 4.2.1, with a 2-sided significance test set at *P* < .05.

## Results

### Demographic and Clinical Characteristics of GDD Cohort

**Figure 1.**
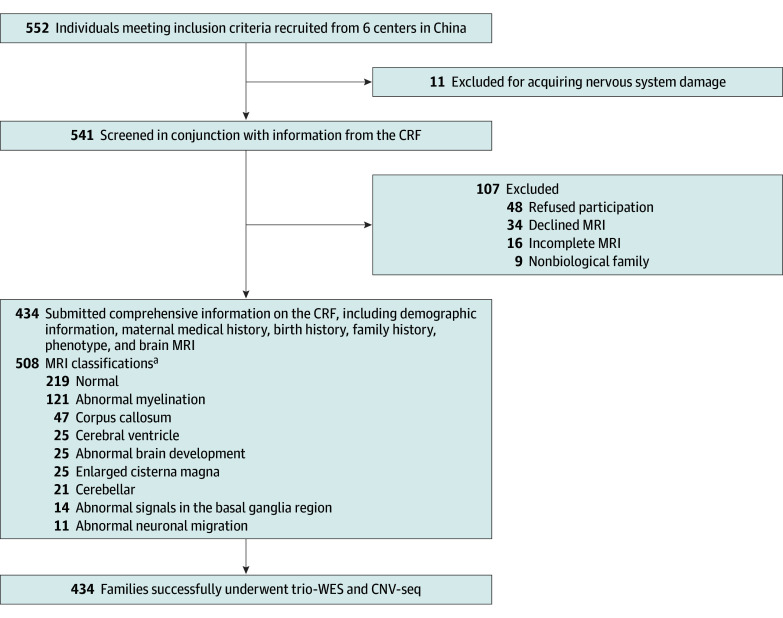
Study Flow Diagram CNV-seq indicates copy number variation sequencing; CRF, case report form; MRI, magnetic resonance imaging; trio-WES, whole exome sequencing. ^a^Some individuals had more than 1 type of classification.

**Figure 2.**
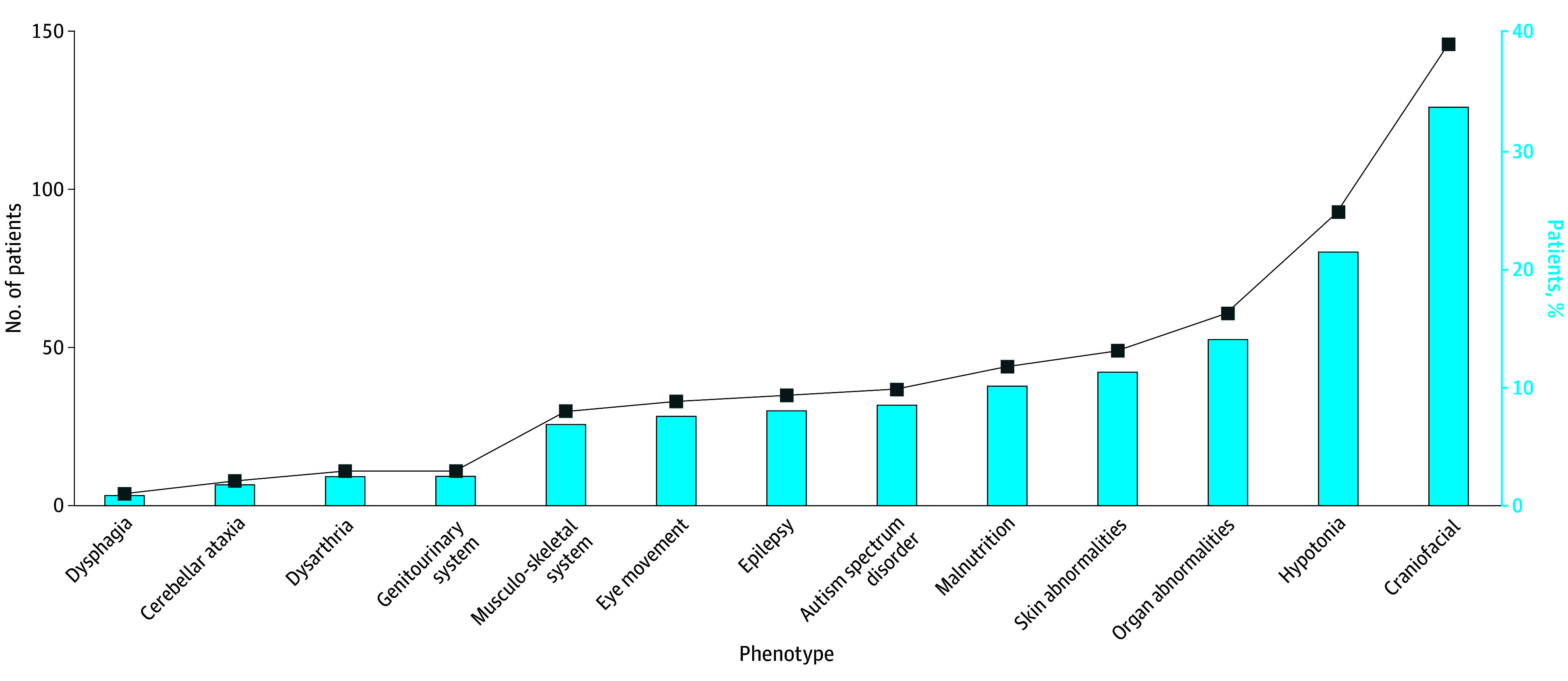
Clinical Phenotypes of the Global Developmental Delay Cohort

In this multicenter, prospective cohort study, a total of 434 children (mean [SD] age, 25.75 [13.24] months) with GDD were included, adhering strictly to the defined inclusion and exclusion criteria. The study workflow is visually represented in Figure 1. Of the participants, 262 (60%) were male and 172 (40%) were female. Most patients (251 [58%]) were younger than 24 months. The study revealed a diverse array of clinical phenotypes among patients with GDD (Figure 2). Alongside cognitive impairment, 146 (34%) exhibited craniofacial abnormalities, and 93 (21%) were affected by hypotonia. Furthermore, organ abnormalities were noted in 61 (14%), and skin abnormalities were present in 49 (11%). All patients underwent brain MRI, with 219 patients (50%) having normal results, 153 (35%) displaying a single type of abnormality, and 62 (14%) showing multiple abnormalities.

### Trio-WES and CNV-Seq Integration 

This study attained a detection rate of 61% (n = 263) through the combined trio-WES and CNV-seq approach (eTable 1 in Supplement 2). Specifically, SNVs and indels were identified with a positive detection rate (defined as the ratio of the sum of pathogenic, likely pathogenic, and uncertain variants to the total number of participants in the genetic testing) of 47% (n = 204) (eFigure 3A in Supplement 1). Pathogenic, likely pathogenic, and uncertain variants were observed in 99, 66, and 39 patients, respectively. Notably, these variants were predominantly located on chromosomes X, 2, and 5 (eFigure 3B in Supplement 1). The most prevalent variant types causing protein alterations included missense, frameshift, and nonsense mutations. In addition, trio-WES revealed 30 CNVs (eFigure 3A in Supplement 1). It linked the *TRIO* (OMIM 601893) variant (NM_007118.4: exon34dup) on chromosome 5 to ID and the *MECP2* (OMIM 30005) variant on the X chromosome to Xq28 duplication syndrome. Notably, these findings were not captured by CNV-seq. The detection rate for trio-WES was 54% (n = 234) (eTable 1 in Supplement 2); further details are available in eTables 2 and 3 in Supplement 2.

CNV-seq successfully identified CNVs in 64 individuals, revealing a positive detection rate of 15% (eFigure 3A in Supplement 1 and eTable 1 in Supplement 2). The preponderance of CNVs was noted on chromosomes 15 and 22. The predominant nature of all CNV segments was characterized by deletions. Specifically, the deletion within the15q11.2-q13.1 region, commonly associated with Angelman syndrome and Prader-Willi syndrome, emerged as the most prevalent. Comprehensive results of the CNV-seq analysis are given in eTable 4 in Supplement 2.

### Correlation of Clinical Phenotypes and Detection in Trio-WES and CNV-Seq

Subsequent analysis delved into the association between clinical features and identified genetic variations. The analysis indicated which findings were more likely to indicate a genetic origin: patients with GDD who were aged 12 to 24 months (164 with positive and 87 with negative vs 99 with positive and 84 with negative genetic test results; *P* = .02), moderate or higher cognitive impairment with a developmental quotient less than 55 (216 with positive and 120 with negative vs 47 with positive and 51 with negative genetic test results; *P* = .005), craniofacial abnormalities (109 with positive and 37 with negative vs 154 with positive and 134 with negative genetic test results; *P* < .001), and multiple MRIs (46 with positive and 16 with negative vs 217 with positive and 155 with negative genetic test results; *P* = .02) (Table). Variables such as sex, family history, adverse pregnancy outcomes, high-risk factors during pregnancy, delivery time, delivery mode, birth asphyxia, musculoskeletal abnormalities, skin abnormalities, organ abnormalities, dysarthria, hypotonia, abnormal eye movements, malnutrition, autism spectrum disorder, and epilepsy did not exhibit a significant association. Craniofacial abnormalities (odds ratio [OR], 2.27; 95% CI, 1.45-3.56), moderate or higher cognitive impairment (OR, 1.69; 95% CI, 1.05-2.70), and age of 12 to 24 months (OR, 1.57; 95% CI, 1.05-2.35) were associated with an increased risk of carrying genetic variants (eFigure 3C in Supplement 1). Additionally, a higher number of phenotypes showed a positive correlation with an increased detection rate, reaching significance with 3 or more phenotypes (3-4 phenotypes: 64 of 94 [68%] with positive genetic test results, *P* = .03; 5-7 phenotypes: 15 of 18 [83%] with positive genetic test results, *P* = .02) (eTable 5 in Supplement 2).

**Table.** Comparison of Genetic Testing Results Across Various Clinical Phenotypes

Characteristic	No. of test results^a^		*P* value (Fisher exact test)
	Positive	Negative	
Sex			
Male	149	113	.06
Female	114	58	
Age group, mo			
12-24	164	87	.02
25-60	99	84	
DQ			
55-75	47	51	.005
<55	216	120	
Family history of GDD			
Yes	37	20	.56
No	226	151	
Adverse pregnancy outcomes			
Yes	10	14	.06
No	253	157	
High-risk pregnancy			
Yes	87	51	.53
No	176	120	
Delivery time			
Premature delivery or postterm delivery	25	16	>.99
Term delivery	238	155	
Delivery mode			
Natural birth	113	73	>.99
Cesarean delivery	150	98	
Asphyxia history			
Yes	22	19	.40
No	241	152	
Craniofacial			
Yes	109	37	<.001
No	154	134	
Abnormality of the musculoskeletal system			
Yes	22	8	.18
No	241	163	
Skin abnormalities			
Yes	35	14	.12
No	228	157	
Organ abnormalities			
Yes	43	18	.09
No	220	153	
Dysarthria			
Yes	4	7	.12
No	259	164	
Hypotonia			
Yes	60	33	.40
No	203	138	
Abnormality of eye movements			
Yes	24	9	.19
No	239	162	
Autism spectrum disorder			
Yes	17	20	.08
No	246	151	
Epilepsy			
Yes	21	14	>.99
No	242	157	
Malnutrition			
Yes	31	13	.19
No	232	158	
MRI			
Normal or abnormal^b^	217	155	.02
Multiple abnormal^c^	46	16	

Abbreviations: DQ, developmental quotient; GDD, global developmental delay; MRI, magnetic resonance imaging.

^a^ Positive results indicate the detection of clinically relevant variants by trio whole exome sequencing or copy number variation sequencing. In contrast, negative results from trio whole exome sequencing and copy number variation sequencing indicate the absence of clinically relevant variants.

^b^ Abnormal indicates the presence of abnormalities in only 1 aspect of MRI.

^c^ Multiple abnormal indicates the presence of abnormalities in multiple aspects of MRI.

### Association Among Genetic Factors, Brain Development, and Clinical Phenotypes

Enrichment analysis of our GDD cohort revealed significant findings, with GO enrichment results reaching significance at an adjusted *P* < .001 (eTable 6 in Supplement 2). The primary areas of enrichment included chromatin (47 of 154), neurons (45 of 154), protein modification (30 of 154), channels (25 of 154), and transcription (12 of 154). Notably, cognitive processes (*ADNP* [OMIM 611386], *AFF2* [OMIM 300806], *DLG4* [OMIM 602887], *EP300* [OMIM 602700], *GRIN1* [OMIM 138249], *GRIN2B* [OMIM 138252], *MECP2* [OMIM 300005], *NEUROD2* [OMIM 601725], *PLA2G6* [OMIM 603604], *SCN2A* [OMIM 182390], *SHROOM4* [OMIM 300579], *SYNGAP1* [OMIM 603384], *TH* [OMIM 191290], *UBE3A* [OMIM 601623], and *VLDLR* [OMIM 192977] [15 of 154]) exhibited significant enrichment as well, with close connections to neuronal function genes (10 of 16), as well as channels (5 of 16), chromatin (4 of 16), and protein modification and transcription processes (2 of 16) (eFigure 4 in Supplement 1). Key cognitive-related genes, such as *SYNGAP1, GRIN2B, GRIN1, DLG4, SCN2A, ADNP, MECP2*, and *EP300,* played central roles in the GDD network (eFigure 5 in Supplement 1), influencing brain development and function and ultimately contributing to the core symptoms of GDD (eFigure 6 in Supplement 1).

### Association Between Dopaminergic Synapse and Cognitive Impairment

**Figure 3.**
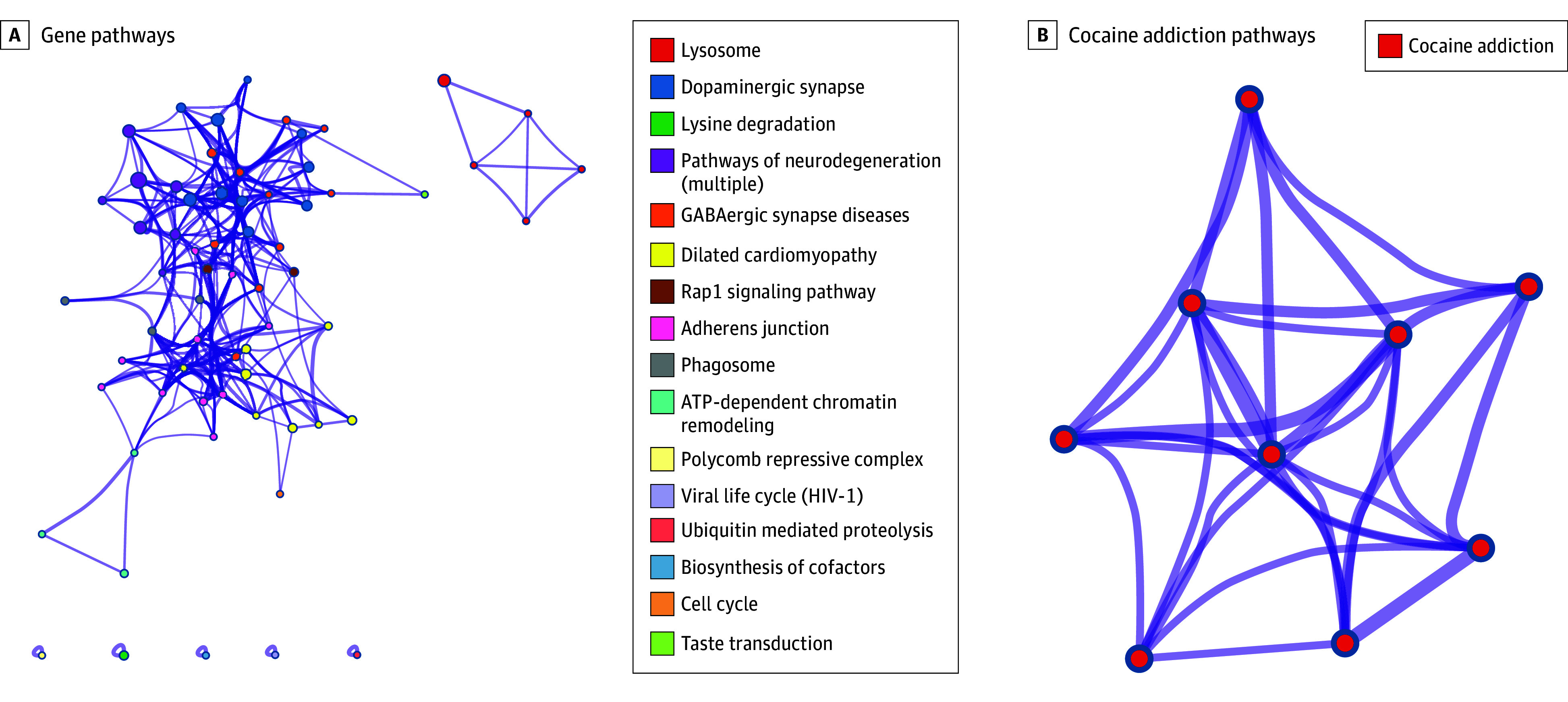
Kyoto Encyclopedia of Genes and Genomes Enrichment Analysis Results The analysis reveals a diverse and intricate network of pathways associated with the genes in the cohort. The pathways are arranged in descending order of significance, with the *P* value gradually increasing. A, Among these pathways, dopaminergic synapses hold the second most prominent position in the entire network. B, The genes associated with cognition exhibit enrichment in the pathway related to cocaine addiction. ATP indicates adenosine triphosphate; GABA, γ-aminobutyric acid.

KEGG pathway enrichment analysis revealed that GDD-related genes were predominantly enriched in lysosome, dopaminergic synapse, and lysine degradation pathways (Figure 3A). Notably, the dopaminergic synapse emerged as the second most crucial pathway associated with GDD. Furthermore, cognition-related genes were primarily linked to cocaine addiction (Figure 3B), a condition connected to dopamine neurotransmitter regulation.^28^

In a subset of 100 randomly selected patients, we measured dopamine concentration. The findings revealed a lower plasma dopamine concentration specifically in patients with severe or profound cognitive impairment (489 [IQR, 344-649] vs 417 [IQR, 322-489] pg/mL [to convert to picomoles per liter, multiply by 6.528]; *P* = .04) (eFigure 7A in Supplement 1). No significant association was observed in the domains of gross motor, fine motor, language, or social function (eFigure 7B-E in Supplement 1). However, there was a general trend toward lower plasma dopamine levels in patients with severe and profound GDD. Although genetic factors alone did not exhibit an association with dopamine concentrations, a noteworthy reduction in dopamine concentration was noted in patients who had both a positive genetic test result (350 [IQR, 309-470] vs 483 [IQR, 347-634] pg/mL, *P* = .046) and a developmental quotient less than 40 (350 [IQR, 309-470] vs 521 [IQR, 357-649], *P* = .03) (eFigure 7F and G in Supplement 1). Importantly, there was no association between sex and dopamine (eFigure 7H in Supplement 1).

## Discussion

This study underscores the significant practical value of combining trio-WES with CNV-seq in the etiologic diagnosis of early childhood GDD. Additionally, we aim to enhance understanding regarding the indications for detection and explore the potential of dopaminergic neurotransmitter as a biomarker and therapeutic target for intervention. To the best of our knowledge, this study represents the largest prospective examination of combined genetic testing methods in a GDD cohort.^15,29,30,31,32^

The thorough and systematic analysis of the GDD cohort’s phenotypes serves to broaden the understanding of the disease’s spectrum and heighten awareness among health care professionals. Genetic studies focusing exclusively on children with GDD are infrequent, with many investigations encompassing a broad age range and displaying incomplete analysis of phenotypic information. In our cohort, the prevalence of comorbid epilepsy (29.6% to 44.0%) and autism spectrum disorder (24.6% to 25.6%) was notably lower than reported in prior studies.^33,34,35,36^ This difference could be attributed to the inclusion of 251 children (58%) aged 12 to 24 months, whose phenotypes may not have fully manifested. Additionally, our cohort contributes supplementary data on the occurrence rates of maternal pregnancy high-risk factors, adverse pregnancy outcomes, skin abnormalities, dysarthria, cerebellar ataxia, and malnutrition. This information forms a foundation for more accurate identification of GDD phenotypes.

The integration of genetic testing has significantly improved the diagnosis rate, reduced missed diagnoses, and clarified the indications for detection. In this study, the combined use of trio-WES with CNV-seq in children with early-stage GDD resulted in a noteworthy positive detection rate of 61%. This integrated approach demonstrated a significant improvement compared with individual testing methods, enhancing the positive detection rate by 18% to 40%.^33,34,35,36,37,38,39,40,41,42,43^ This approach not only saved time for families but also reduced economic costs that would have been incurred through trying different testing methods.^44,45^ Moreover, the combination of trio-WES and CNV-seq testing addressed the limitations of each method, leading to a more comprehensive genetic analysis^15^ and a reduction in missed diagnoses. Due to technological constraints, trio-WES may miss 55% of CNV variations, whereas CNV-seq has a missed diagnosis rate of 3%. Of note, CNV-seq cannot detect SNVs or indels.^46^ Finally, despite the clinical heterogeneity of GDD phenotypes, strict adherence to indications for genetic testing is crucial to avoid unnecessary medical interventions. Currently, there exists a lack of clarity among health care professionals regarding the necessity of genetic testing.^47^ Previous studies have indicated a correlation between positive genetic testing rates and factors such as family history, male sex, and number of phenotypes.^48,49,50^ In our study, we have further refined the indications for testing, establishing that patients aged 12 to 24 months and those with moderate or higher cognitive impairment, craniofacial abnormalities, or complex phenotypes are more likely candidates for genetic testing. We observed a slightly increased prevalence of craniofacial abnormalities reported in Eastern countries compared with Western countries, possibly due to geographic variation.^34,36,51^ Moreover, the increased rate of positive genetic test results among children aged 12 to 24 months compared with older children may be influenced by the age distribution within this specific cohort.

Understanding the intricate connection among genetic variation, brain development, and phenotypes is crucial for enhancing our comprehension of the mechanisms underlying GDD. Brain development is a meticulously regulated process, encompassing the proliferation and migration of progenitor cells, the maturation of neurons, and the formation of neural circuits, all of which are under the control of the human genome. The precise mechanisms by which the genome influences cognitive function and brain development are still under investigation.^52^ Many genes associated with GDD are predominantly expressed in early life,^53^ impacting early nervous system development. Research has demonstrated that genes linked to GDD and ID are enriched in 309 GO terms and 59 KEGG pathways, primarily related to neural development, cellular processes, and metabolism.^29^ Our cohort study underscores the paramount importance of cognitive processes in the pathogenesis of GDD. Notably, genes such as *SYNGAP1, GRIN2B, GRIN1, DLG4, SCN2A, ADNP, MECP2*, and *EP300*, despite their diverse biological functions, all contribute to abnormal brain development and function, elucidating the core symptoms of GDD. The close association among genetic variation, brain development, and phenotype explains the pathogenesis in numerous GDD cases.

The dopamine neurotransmitter has been identified as being associated with the severity of cognitive impairment and holds potential as a new target for precision medicine. Previous studies have highlighted intervention targets, such as γ-aminobutyric acid receptors and sodium and calcium channels.^54,55,56^ Additionally, gene editing technology has been used to restore gene function and modify brain development.^57^ In this cohort study, our findings support the correlation between dopaminergic synapse and cognitive impairment, as substantiated by prior research and animal models.^58,59,60^ Therefore, targeting the dopaminergic pathway holds promise for treating GDD and ID, although further comprehensive research is needed to confirm the efficacy of this approach.

### Limitations

This study has several limitations that warrant consideration. First, the findings are based on an early childhood GDD cohort; thus, caution is needed when extrapolating the results to other populations. Second, potential differences may exist between the clinical phenotypes collected in this cohort and those in national or global GDD cohorts. Further exploration is required to determine the applicability of the indications for detection to other cohorts. Lastly, our study included only a subset of patients with GDD for plasma dopamine detection. Expanding the sample size and conducting in vivo and in vitro experiments are necessary steps to verify whether dopamine can be targeted for clinical precision medical intervention in patients with GDD. This study serves as an initial exploration, presenting a new research direction for intervention therapy in GDD.

## Conclusions

This study supports the clinical utility of combining trio-WES sequencing with CNV-seq in the etiologic diagnosis and clinical management of early-childhood GDD. Moreover, we have identified an association among genetic variation, brain development, and clinical phenotype. Notably, our findings also suggest a positive correlation exists between dopaminergic synapse and GDD.

## Notes

Supplement 1. **eMethods.** Supplementary Methods **eFigure 1.** Flowchart for Analyzing SNVs and Indels Data Using Trio-WES **eFigure 2.** Flowchart for Analyzing CNVs Using Trio-WES and CNV-Seq **eFigure 3.** Analysis of Trio-WES+CNV-Seq Sequencing Results **eFigure 4.** GO Enrichment Analysis Results **eFigure 5.** Protein Interaction Networks Utilized to Identify Hub Genes **eFigure 6.** Genetic Variations-Brain Development-Phenotypic Relationship Network **eFigure 7.** DA Levels
eReferences

Supplement 2. **eTable 1.** Comparison of the number of clinically-relevant variants found by different detection methods **eTable 2.** SNVs/Indels detected by trio-WES **eTable 3.** CNVs detected by trio-WES **eTable 4.** CNVs detected by CNV-seq **eTable 5.** The connection between the number of phenotypes and the number of variants **eTable 6.** GO enrichment analysis results

Supplement 3.
Data Sharing Statement

## References

[1] Maulik PK, Mascarenhas MN, Mathers CD, Dua T, Saxena S. Prevalence of intellectual disability: a meta-analysis of population-based studies. Res Dev Disabil. 2011;32(2):419-436. doi:10.1016/j.ridd.2010.12.018 21236634

[2] Tremblay I, Janvier A, Laberge AM. Paediatricians underuse recommended genetic tests in children with global developmental delay. Paediatr Child Health. 2018;23(8):e156-e162. doi:10.1093/pch/pxy033 30842697 PMC6241916

[3] Shashi V, McConkie-Rosell A, Rosell B, . The utility of the traditional medical genetics diagnostic evaluation in the context of next-generation sequencing for undiagnosed genetic disorders. Genet Med. 2014;16(2):176-182. doi:10.1038/gim.2013.99 23928913

[4] GBD 2019 Mental Disorders Collaborators. Global, regional, and national burden of 12 mental disorders in 204 countries and territories, 1990-2019: a systematic analysis for the Global Burden of Disease Study 2019. Lancet Psychiatry. 2022;9(2):137-150. doi:10.1016/S2215-0366(21)00395-3 35026139 PMC8776563

[5] van Wassenaer-Leemhuis A. Parental report of early cognitive development: benefits, and next steps. Lancet Child Adolesc Health. 2019;3(10):666-668. doi:10.1016/S2352-4642(19)30190-7 31402195

[6] Jeong J, Franchett EE, Ramos de Oliveira CV, Rehmani K, Yousafzai AK. Parenting interventions to promote early child development in the first three years of life: a global systematic review and meta-analysis. PLoS Med. 2021;18(5):e1003602. doi:10.1371/journal.pmed.1003602 33970913 PMC8109838

[7] Malinowski J, Miller DT, Demmer L, ; ACMG Professional Practice and Guidelines Committee. Systematic evidence-based review: outcomes from exome and genome sequencing for pediatric patients with congenital anomalies or intellectual disability. Genet Med. 2020;22(6):986-1004. doi:10.1038/s41436-020-0771-z 32203227 PMC7222126

[8] Srivastava AK, Schwartz CE. Intellectual disability and autism spectrum disorders: causal genes and molecular mechanisms. Neurosci Biobehav Rev. 2014;46(pt 2):161-174. doi:10.1016/j.neubiorev.2014.02.015 24709068 PMC4185273

[9] Karaca E, Harel T, Pehlivan D, . Genes that affect brain structure and function identified by rare variant analyses of mendelian neurologic disease. Neuron. 2015;88(3):499-513. doi:10.1016/j.neuron.2015.09.048 26539891 PMC4824012

[10] Anazi S, Maddirevula S, Faqeih E, . Clinical genomics expands the morbid genome of intellectual disability and offers a high diagnostic yield. Mol Psychiatry. 2017;22(4):615-624. doi:10.1038/mp.2016.113 27431290

[11] Mitani T, Isikay S, Gezdirici A, ; Baylor-Hopkins Center for Mendelian Genomics. High prevalence of multilocus pathogenic variation in neurodevelopmental disorders in the Turkish population. Am J Hum Genet. 2021;108(10):1981-2005. doi:10.1016/j.ajhg.2021.08.009 34582790 PMC8546040

[12] Cortese S, Solmi M, Michelini G, . Candidate diagnostic biomarkers for neurodevelopmental disorders in children and adolescents: a systematic review. World Psychiatry. 2023;22(1):129-149. doi:10.1002/wps.21037 36640395 PMC9840506

[13] Siegel M, McGuire K, Veenstra-VanderWeele J, ; American Academy of Child and Adolescent Psychiatry (AACAP) Committee on Quality Issues (CQI). Practice parameter for the assessment and treatment of psychiatric disorders in children and adolescents with intellectual disability (intellectual developmental disorder). J Am Acad Child Adolesc Psychiatry. 2020;59(4):468-496. doi:10.1016/j.jaac.2019.11.018 33928910

[14] Vissers LE, Gilissen C, Veltman JA. Genetic studies in intellectual disability and related disorders. Nat Rev Genet. 2016;17(1):9-18. doi:10.1038/nrg3999 26503795

[15] Srivastava S, Love-Nichols JA, Dies KA, ; NDD Exome Scoping Review Work Group. Meta-analysis and multidisciplinary consensus statement: exome sequencing is a first-tier clinical diagnostic test for individuals with neurodevelopmental disorders. Genet Med. 2019;21(11):2413-2421. doi:10.1038/s41436-019-0554-6 31182824 PMC6831729

[16] Martin CL, Ledbetter DH. Chromosomal microarray testing for children with unexplained neurodevelopmental disorders. JAMA. 2017;317(24):2545-2546. doi:10.1001/jama.2017.7272 28654998 PMC7058144

[17] Wright CF, FitzPatrick DR, Firth HV. Paediatric genomics: diagnosing rare disease in children. Nat Rev Genet. 2018;19(5):253-268. doi:10.1038/nrg.2017.116 29398702

[18] Duan J, Zhang JG, Deng HW, Wang YP. Comparative studies of copy number variation detection methods for next-generation sequencing technologies. PLoS One. 2013;8(3):e59128. doi:10.1371/journal.pone.0059128 23527109 PMC3604020

[19] Chen X, Jiang Y, Chen R, . Clinical efficiency of simultaneous CNV-seq and whole-exome sequencing for testing fetal structural anomalies. J Transl Med. 2022;20(1):10. doi:10.1186/s12967-021-03202-9 34980134 PMC8722033

[20] Quach TT, Stratton HJ, Khanna R, . Intellectual disability: dendritic anomalies and emerging genetic perspectives. Acta Neuropathol. 2021;141(2):139-158. doi:10.1007/s00401-020-02244-5 33226471 PMC7855540

[21] Zoghbi HY, Bear MF. Synaptic dysfunction in neurodevelopmental disorders associated with autism and intellectual disabilities. Cold Spring Harb Perspect Biol. 2012;4(3):a009886. doi:10.1101/cshperspect.a009886 22258914 PMC3282414

[22] Fernández-Blanco Á, Dierssen M. Rethinking intellectual disability from neuro- to astro-pathology. Int J Mol Sci. 2020;21(23):9039. doi:10.3390/ijms21239039 33261169 PMC7730506

[23] Di Marco B, Bonaccorso CM, Aloisi E, D’Antoni S, Catania MV. Neuro-inflammatory mechanisms in developmental disorders associated with intellectual disability and autism spectrum disorder: a neuro- immune perspective. CNS Neurol Disord Drug Targets. 2016;15(4):448-463. doi:10.2174/1871527315666160321105039 26996174

[24] Burack JA, Evans DW, Russo N, Napoleon JS, Goldman KJ, Iarocci G. Developmental perspectives on the study of persons with intellectual disability. Annu Rev Clin Psychol. 2021;17:339-363. doi:10.1146/annurev-clinpsy-081219-090532 33561363

[25] Zhou Y, Zhou B, Pache L, . Metascape provides a biologist-oriented resource for the analysis of systems-level datasets. Nat Commun. 2019;10(1):1523. doi:10.1038/s41467-019-09234-6 30944313 PMC6447622

[26] Szklarczyk D, Gable AL, Lyon D, . STRING v11: protein-protein association networks with increased coverage, supporting functional discovery in genome-wide experimental datasets. Nucleic Acids Res. 2019;47(D1):D607-D613. doi:10.1093/nar/gky1131 30476243 PMC6323986

[27] Shannon P, Markiel A, Ozier O, . Cytoscape: a software environment for integrated models of biomolecular interaction networks. Genome Res. 2003;13(11):2498-2504. doi:10.1101/gr.1239303 14597658 PMC403769

[28] Danielsson K, Stomberg R, Adermark L, Ericson M, Söderpalm B. Differential dopamine release by psychosis-generating and non-psychosis-generating addictive substances in the nucleus accumbens and dorsomedial striatum. Transl Psychiatry. 2021;11(1):472. doi:10.1038/s41398-021-01589-z 34518523 PMC8438030

[29] Sánchez-Luquez KY, Carpena MX, Karam SM, Tovo-Rodrigues L. The contribution of whole-exome sequencing to intellectual disability diagnosis and knowledge of underlying molecular mechanisms: a systematic review and meta-analysis. Mutat Res Rev Mutat Res. 2022;790:108428. doi:10.1016/j.mrrev.2022.108428 35905832

[30] Retterer K, Juusola J, Cho MT, . Clinical application of whole-exome sequencing across clinical indications. Genet Med. 2016;18(7):696-704. doi:10.1038/gim.2015.148 26633542

[31] Chen S, Xiong J, Chen B, . Autism spectrum disorder and comorbid neurodevelopmental disorders (ASD-NDDs): clinical and genetic profile of a pediatric cohort. Clin Chim Acta. 2022;524:179-186. doi:10.1016/j.cca.2021.11.014 34800434

[32] Kipkemoi P, Kim HA, Christ B, ; NeuroDev Project. Phenotype and genetic analysis of data collected within the first year of NeuroDev. Neuron. 2023;111(18):2800-2810.e5. doi:10.1016/j.neuron.2023.06.010 37463579

[33] Zhang H, Chen X, Tan H, . The exploration of genetic aetiology and diagnostic strategy for 321 Chinese individuals with intellectual disability. Clin Chim Acta. 2023;538:94-103. doi:10.1016/j.cca.2022.10.023 36368352

[34] Chen JS, Yu WH, Tsai MC, Hung PL, Tu YF. Comorbidities associated with genetic abnormalities in children with intellectual disability. Sci Rep. 2021;11(1):6563. doi:10.1038/s41598-021-86131-3 33753861 PMC7985145

[35] Hiraide T, Yamoto K, Masunaga Y, . Genetic and phenotypic analysis of 101 patients with developmental delay or intellectual disability using whole-exome sequencing. Clin Genet. 2021;100(1):40-50. doi:10.1111/cge.13951 33644862

[36] Seo GH, Lee H, Lee J, . Diagnostic performance of automated, streamlined, daily updated exome analysis in patients with neurodevelopmental delay. Mol Med. 2022;28(1):38. doi:10.1186/s10020-022-00464-x 35346031 PMC8962085

[37] Global Research on Developmental Disabilities Collaborators. Developmental disabilities among children younger than 5 years in 195 countries and territories, 1990-2016: a systematic analysis for the Global Burden of Disease Study 2016. Lancet Glob Health. 2018;6(10):e1100-e1121. doi:10.1016/S2214-109X(18)30309-7 30172774 PMC6139259

[38] Deciphering Developmental Disorders Study. Large-scale discovery of novel genetic causes of developmental disorders. Nature. 2015;519(7542):223-228. doi:10.1038/nature14135 25533962 PMC5955210

[39] Cieza A, Causey K, Kamenov K, Hanson SW, Chatterji S, Vos T. Global estimates of the need for rehabilitation based on the Global Burden of Disease study 2019: a systematic analysis for the Global Burden of Disease Study 2019. Lancet. 2021;396(10267):2006-2017. doi:10.1016/S0140-6736(20)32340-0 33275908 PMC7811204

[40] Fell CW, Nagy V. Cellular models and high-throughput screening for genetic causality of intellectual disability. Trends Mol Med. 2021;27(3):220-230. doi:10.1016/j.molmed.2020.12.003 33397633

[41] Raznahan A, Won H, Glahn DC, Jacquemont S. Convergence and divergence of rare genetic disorders on brain phenotypes: a review. JAMA Psychiatry. 2022;79(8):818-828. doi:10.1001/jamapsychiatry.2022.1450 35767289

[42] Mollon J, Almasy L, Jacquemont S, Glahn DC. The contribution of copy number variants to psychiatric symptoms and cognitive ability. Mol Psychiatry. 2023;28(4):1480-1493. doi:10.1038/s41380-023-01978-4 36737482 PMC10213133

[43] Wright CF, Fitzgerald TW, Jones WD, ; DDD study. Genetic diagnosis of developmental disorders in the DDD study: a scalable analysis of genome-wide research data. Lancet. 2015;385(9975):1305-1314. doi:10.1016/S0140-6736(14)61705-0 25529582 PMC4392068

[44] Manickam K, McClain MR, Demmer LA, ; ACMG Board of Directors. Exome and genome sequencing for pediatric patients with congenital anomalies or intellectual disability: an evidence-based clinical guideline of the American College of Medical Genetics and Genomics (ACMG). Genet Med. 2021;23(11):2029-2037. doi:10.1038/s41436-021-01242-6 34211152

[45] Doble B, Schofield D, Evans CA, . Impacts of genomics on the health and social costs of intellectual disability. J Med Genet. 2020;57(7):479-486. doi:10.1136/jmedgenet-2019-106445 31980565

[46] Xiao B, Ye X, Wang L, . Whole genome low-coverage sequencing concurrently detecting copy number variations and their underlying complex chromosomal rearrangements by systematic breakpoint mapping in intellectual deficiency/developmental delay patients. Front Genet. 2020;11:616. doi:10.3389/fgene.2020.00616 32733533 PMC7357533

[47] Callahan KP, Feudtner C. Genetic testing is messier in practice than in theory: lessons from neonatology. Am J Bioeth. 2022;22(2):37-39. doi:10.1080/15265161.2021.2013978 35089833 PMC8936853

[48] Spataro N, Trujillo-Quintero JP, Manso C, . High performance of a dominant/X-linked gene panel in patients with neurodevelopmental disorders. Genes (Basel). 2023;14(3):708. doi:10.3390/genes14030708 36980980 PMC10048137

[49] Levchenko O, Dadali E, Bessonova L, . Complex diagnostics of non-specific intellectual developmental disorder. Int J Mol Sci. 2022;23(14):7764. doi:10.3390/ijms23147764 35887114 PMC9323143

[50] Lichtenstein P, Tideman M, Sullivan PF, . Familial risk and heritability of intellectual disability: a population-based cohort study in Sweden. J Child Psychol Psychiatry. 2022;63(9):1092-1102. doi:10.1111/jcpp.13560 34921396

[51] Bowling KM, Thompson ML, Amaral MD, . Genomic diagnosis for children with intellectual disability and/or developmental delay. Genome Med. 2017;9(1):43. doi:10.1186/s13073-017-0433-1 28554332 PMC5448144

[52] Kelley KW, Pașca SP. Human brain organogenesis: toward a cellular understanding of development and disease. Cell. 2022;185(1):42-61. doi:10.1016/j.cell.2021.10.003 34774127 PMC13523617

[53] Li N, Zhou P, Tang H, . In-depth analysis reveals complex molecular aetiology in a cohort of idiopathic cerebral palsy. Brain. 2022;145(1):119-141. doi:10.1093/brain/awab209 34077496 PMC8967106

[54] Braat S, Kooy RF. The GABAA receptor as a therapeutic target for neurodevelopmental disorders. Neuron. 2015;86(5):1119-1130. doi:10.1016/j.neuron.2015.03.042 26050032

[55] Meisler MH, Hill SF, Yu W. Sodium channelopathies in neurodevelopmental disorders. Nat Rev Neurosci. 2021;22(3):152-166. doi:10.1038/s41583-020-00418-4 33531663 PMC8710247

[56] Kessi M, Chen B, Peng J, Yan F, Yang L, Yin F. Calcium channelopathies and intellectual disability: a systematic review. Orphanet J Rare Dis. 2021;16(1):219. doi:10.1186/s13023-021-01850-0 33985586 PMC8120735

[57] Qian J, Guan X, Xie B, . Multiplex epigenome editing of *MECP2* to rescue Rett syndrome neurons. Sci Transl Med. 2023;15(679):eadd4666. doi:10.1126/scitranslmed.add4666 36652535 PMC11975455

[58] Boot E, Hollak CEM, Huijbregts SCJ, . Cerebral dopamine deficiency, plasma monoamine alterations and neurocognitive deficits in adults with phenylketonuria. Psychol Med. 2017;47(16):2854-2865. doi:10.1017/S0033291717001398 28552082

[59] Klein M, Singgih EL, van Rens A, . Contribution of intellectual disability-related genes to ADHD risk and to locomotor activity in *Drosophila.* Am J Psychiatry. 2020;177(6):526-536. doi:10.1176/appi.ajp.2019.18050599 32046534

[60] Ke Y, Weng M, Chhetri G, . Trappc9 deficiency in mice impairs learning and memory by causing imbalance of dopamine D1 and D2 neurons. Sci Adv. 2020;6(47):eabb7781. doi:10.1126/sciadv.abb7781 33208359 PMC7673810

