# Supplementary material for: Genetic Testing for Global Developmental Delay in Early Childhood
Source: JAMA Netw Open. 2024 Jun 5;7(6):e2415084. doi: 10.1001/jamanetworkopen.2024.15084 (PMC11154162; doi:10.1001/jamanetworkopen.2024.15084)
Supplement: Supplement 1. — eMethods. Supplementary Methods eFigure 1. Flowchart for Analyzing SNVs and Indels Data Using Trio-WES eFigure 2. Flowchart for Analyzing CNVs Using Trio-WES and CNV-Seq eFigure 3. Analysis of Trio-WES+CNV-Seq Sequencing Results eFigure 4. GO Enrichment Analysis Results eFigure 5. Protein Interaction Networks Utilized to Identify Hub Genes eFigure 6. Genetic Variations-Brain Development-Phenotypic Relationship Network eFigure 7. DA Levels eReferences [file jamanetwopen-e2415084-s001.pdf]

# Supplementary Online Content

Zhang J, Xu Y, Liu Y, et al. Genetic testing for global developmental delay in early childhood. *JAMA Netw Open*. 2024;7(6):e2415084.
doi:10.1001/jamanetworkopen.2024.15084

**eMethods.** Supplementary Methods
**eFigure 1.** Flowchart for Analyzing SNVs and Indels Data Using Trio-WES
**eFigure 2.** Flowchart for Analyzing CNVs Using Trio-WES and CNV-Seq
**eFigure 3.** Analysis of Trio-WES+CNV-Seq Sequencing Results
**eFigure 4.** GO Enrichment Analysis Results
**eFigure 5.** Protein Interaction Networks Utilized to Identify Hub Genes
**eFigure 6.** Genetic Variations-Brain Development-Phenotypic Relationship Network
**eFigure 7.** DA Levels
**eReferences**

This supplementary material has been provided by the authors to give readers additional information about their work.

© 2024 Zhang J et al. *JAMA Network Open.*

**eMethods.** Supplementary Methods

**Analysis and data visualization methods in MRI**

The MRI examinations were conducted using the GE SIGNA Pioneer 3.0T MRI scanner with standard head coils for both transmission and reception. The head of the patient was immobilized using a sponge pad. Due to challenges associated with cooperation of the child during the extended MRI procedure, we administered nasal sedation with dexmedetomidine hydrochloride with the informed consent of the legal guardian(s). All participants underwent a standard brain MRI protocol, which included axial T1-weighted imaging, T2-weighted imaging, and sagittal T2-weighted imaging. The scans were performed by a specialized technician under the supervision of a radiologist to monitor and assess image quality in real-time. In cases of motion artifacts, and if feasible, the scans were repeated. Samples with consistently suboptimal image quality were excluded from the analysis. Following the imaging session, the acquired images were interpreted by experienced radiologists, who had undergone standardized training and wrote the reports. Based on the findings, the images were categorized into nine distinct groups. ①Normal: no abnormal MRI findings. ②Abnormal myelination: dysmyelination, delayed myelination, white matter demyelination, myelination disorder. ③ Corpus callosum: partial agenesis of the corpus callosum, agenesia corporis callosi, absence of corpus callosum. ④Cerebral ventricle：abnormal cerebral ventricle morphology. ⑤ Abnormal brain development: cerebral hypoplasia, hydrocephalus. ⑥ Enlarged cisterna magna. ⑦ Cerebellar: cerebellar atrophy, cerebellar dysplasia. ⑧ Abnormal signals in the basal ganglia region：abnormal basal ganglia MRI signal intensity; ⑨ Abnormal neuronal migration: focal cortical dysplasia, gray matter heterotopia, polymicrogyria, microgyria.

**Trio-WES**

The samples underwent sequencing using the IDT xGen Exome Research Panel capture library, followed by Illumina base NovaSeq 6000 or 2500 sequencing technology. Achieving an average sequencing depth of 150X with a Q30>90%. The NGS raw fastq data were aligned to the human reference genome (GRCh38/hg38) using the Burrows–Wheeler Aligner. Our SNV called data set was annotated by ANNOVAR and filtered to retain only high-quality rare SNVs with a potential damaging effect. Specifically, SNVs in coding or exon-intron junctions, with a minor allele frequency (MAF) ≤0.01 compared to gnomAD, ExAC and other public databases were retained. Then variants were classified according to inheritance pattern: de novo variants, autosomal recessive (AR) inheritance of homozygous variants, AR inheritance of compound heterozygous variants, X-linked inheritance. Variants conforming to disease genetic pattern and predicted as deleterious multiple software tools were then selected. According to the guidelines published by the American College of Medical Genetics and Genomics (ACMG), the evaluation of genetic variations is conducted by considering the type of variation, clinical evidence, and procedural guidelines. The ACMG pathogenicity scoring system is employed to classify and score genetic variations based on different types of evidence (such as pathogenic evidence, clinical data, functional experiments, etc.). The ACMG pathogenicity scoring system is employed to classify and score genetic variations based on different types of evidence (such as pathogenic evidence, clinical data, functional experiments, etc.). Pathogenic variants associated with the clinical phenotype of the children were further verified by the Sanger sequencing. The detection process of CNV by Trio-WES was consistent with that of CNV-seq.

**CNV-seq**

The TruSeq Library Building Kit was employed to generate sequencing libraries following the fragmentation of genomic DNA. Subsequently, high-throughput sequencing technology (Illumina, San

© 2024 Zhang J et al. *JAMA Network Open.*

Diego, CA, USA) was utilized to sequence the libraries. The Burrows-Wheeler technique was applied to align all sequences to the human reference genome hg38. Putative CNVs identified in WES data or CNC-seq were selected using the Database of Genomic Variants (http://dgv.tcag.ca). Annotation of CNVs involved referencing public databases (Decipher, ClinVar, ClinGen, ISCA, and dbVar), along with literature reviews. Pathogenicity screening was conducted using databases such as OMIM, DECIPHER, Orphanet, and others.

**KEGG**

To further capture the relationships between the terms, a subset of enriched terms was selected and rendered as a network plot, where terms with a similarity > 0.3 are connected by edges. Optimal terms, based on p-values, were chosen from each of the 20 clusters, ensuring no more than 15 terms per cluster and a total of 250 terms. The resulting network was visualized using Cytoscape, where each node represents an enriched term and colored-coded by its cluster ID.

**ELISA**

The DA antigen was immobilized on the plate labeled with an enzyme. During the experiment, the DA in the sample or the standard product competed with the immobilized DA for binding to the biotin-labeled monoclonal antibody specific for DA. Optical density values were measured at a wavelength of 450nm using an enzyme reader. The concentration of DA in the sample was determined by constructing a standard curve with a coefficient of determination ($R^2$) greater than or equal to 0.99.

© 2024 Zhang J et al. *JAMA Network Open.*

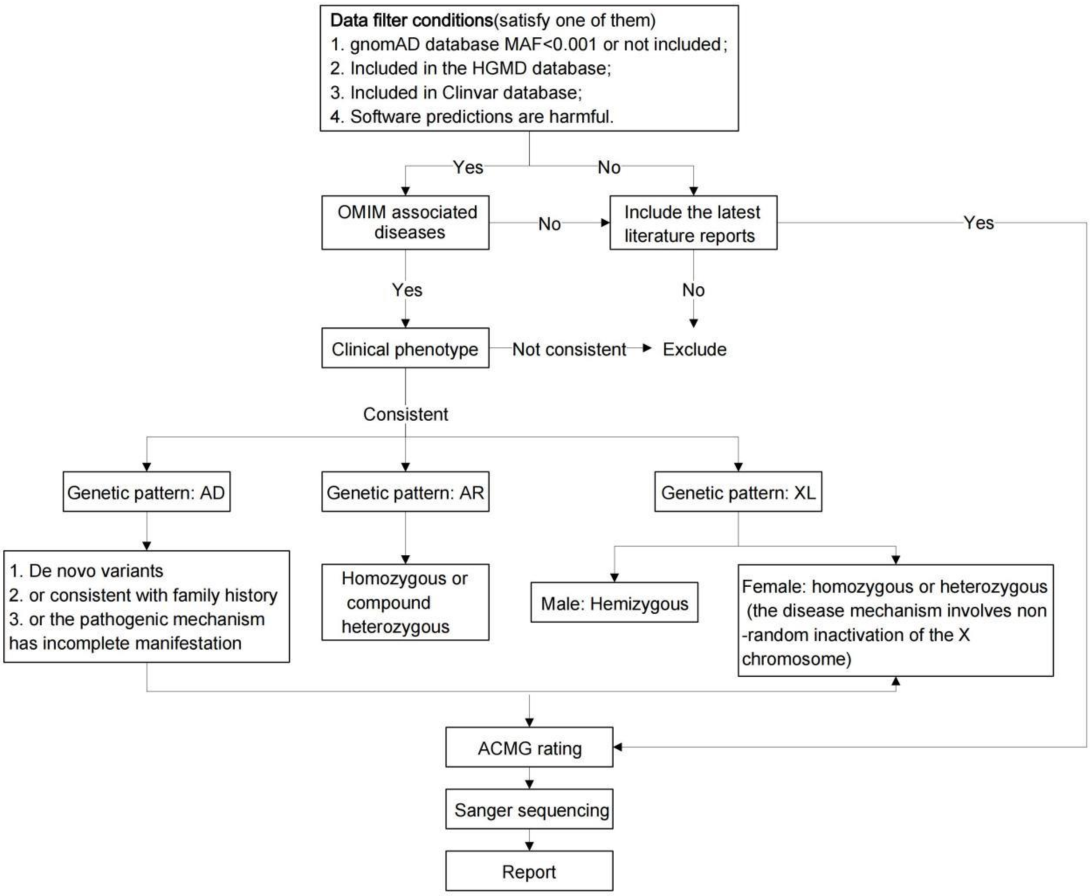


**eFigure 1. Flowchart for analyzing SNVs and Indels data using trio-WES.**

© 2024 Zhang J et al. *JAMA Network Open.*

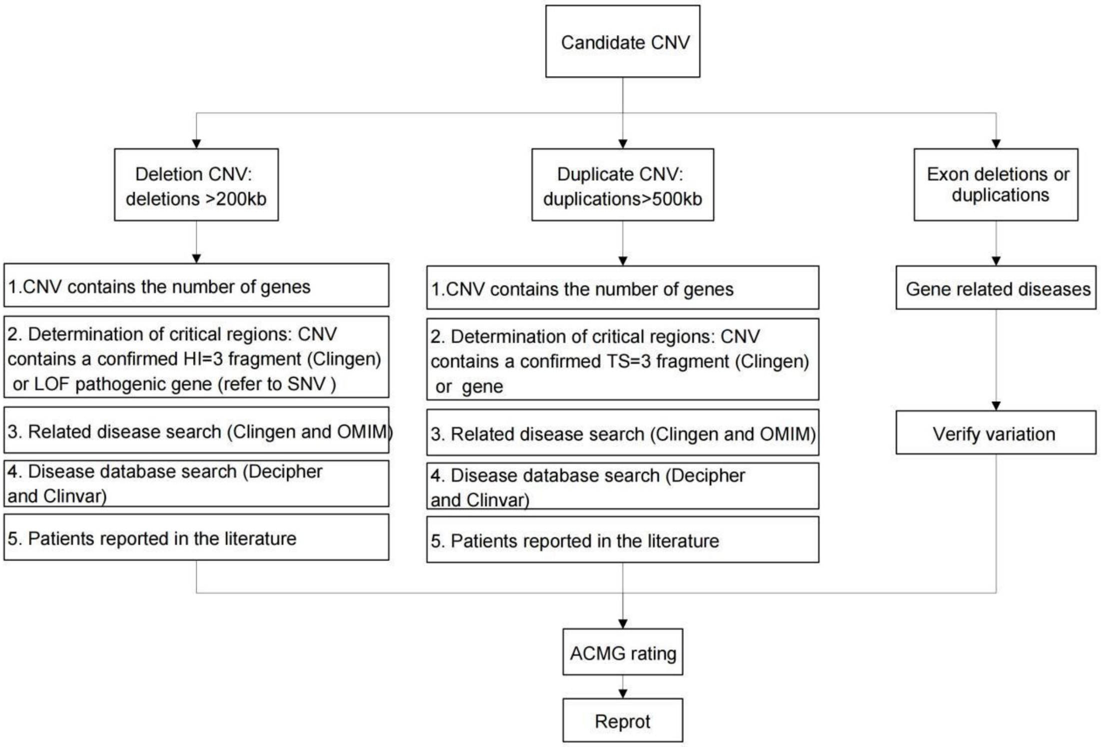


**eFigure 2. Flowchart for analyzing CNVs using trio-WES and CNV-seq.**

© 2024 Zhang J et al. *JAMA Network Open.*

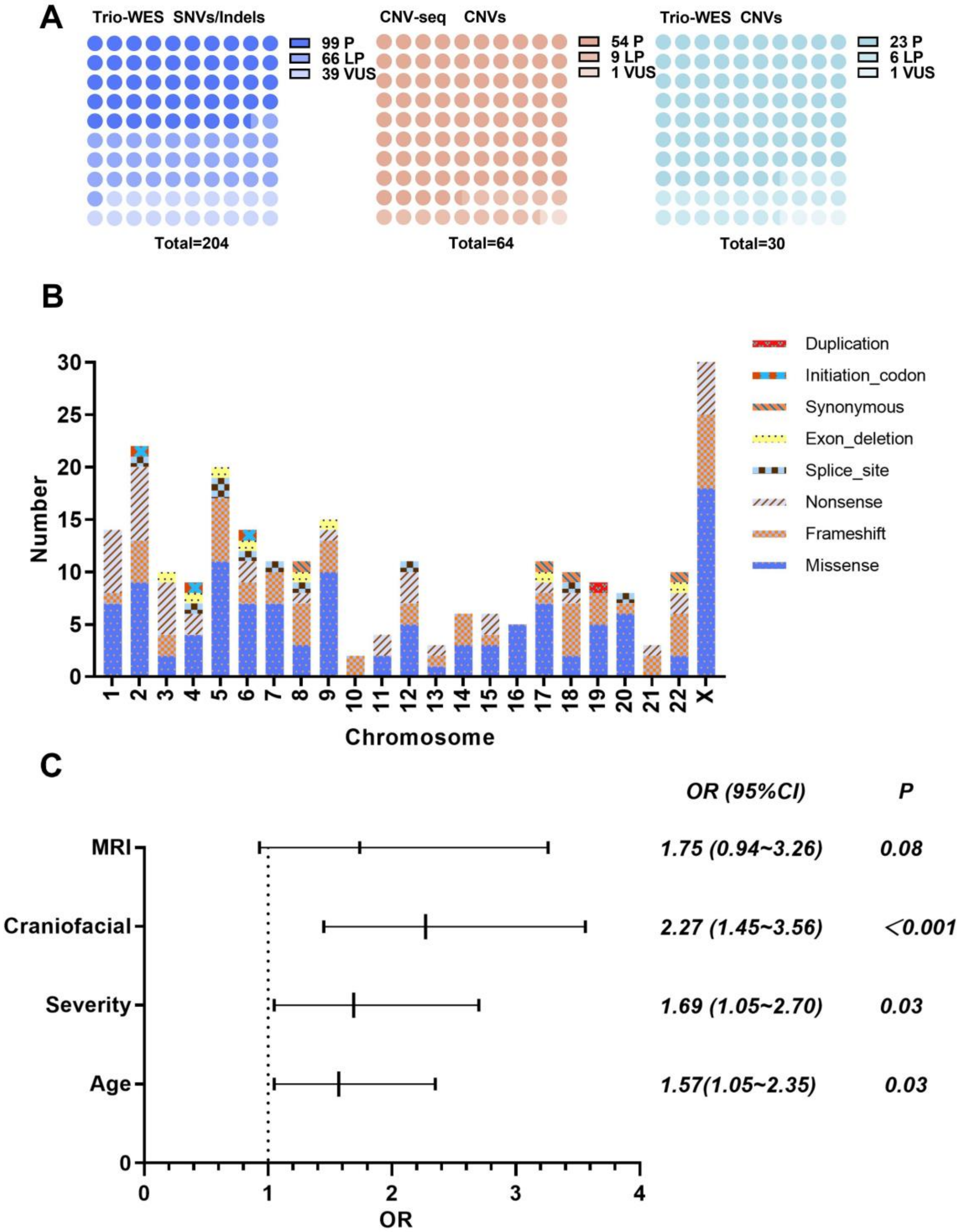


**eFigure 3. Analysis of trio-WES+CNV-seq sequencing results. (A)** The blue color represents SNVs and Indels detected through trio-WES (Left), while the light blue color represents CNVs (Right). The orange color represents CNVs detected through CNV-seq (Center). **(B)** The distribution of SNVs/Indels across the chromosomes. **(C)** Phenotypic analysis related to genetic risk factors in patients. Craniofacial features, disease severity, and patient age were identified as independent risk factors for genetic causative factors.

© 2024 Zhang J et al. *JAMA Network Open.*

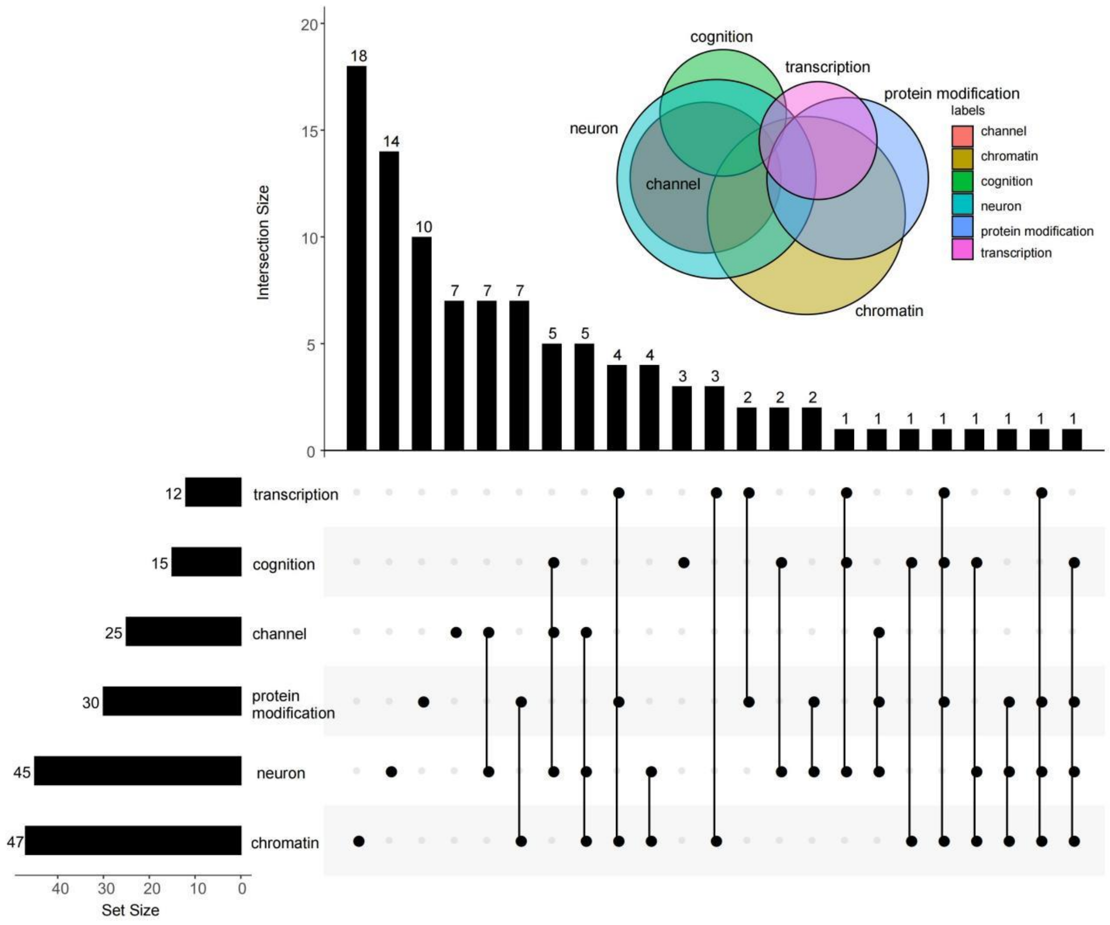


**eFigure 4. GO enrichment analysis results.** Notably, 47 genes were significantly enriched in chromatin-related processes, while 45 genes were associated with neuronal functions. Additionally, 30 genes were involved in protein modification, 25 genes were related to channels, 12 genes were associated with transcription, and 15 genes were implicated in cognitive processes. Notably, the genes associated with cognition intersected with each of the enrichment pathways, emphasizing the pivotal role of cognitive related genes in the pathogenesis of GDD.

© 2024 Zhang J et al. *JAMA Network Open.*

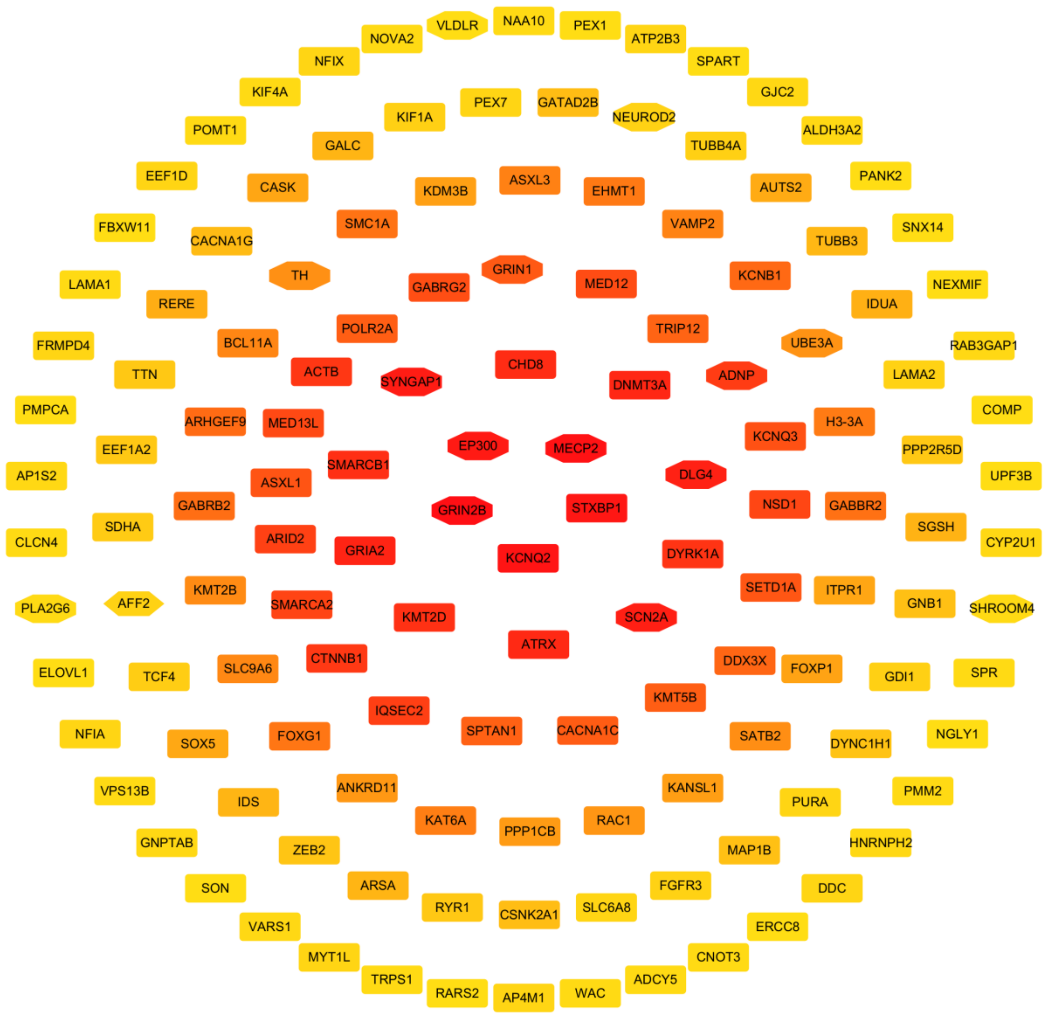


**eFigure 5. Protein interaction networks utilized to identify hub genes.** Notably, the genes associated with cognition, namely *SYNGAP1, GRIN2B, GRIN1, DLG4, SCN2A, ADNP, MECP2,* and *EP300*, occupy crucial positions within the entire network. Employing the "cytoHubba" plugin and the MCC method, key genes were identified through comprehensive network screening, revealing that the aforementioned genes reside in the top quartile of the network. The hexagon represents cognitive-related genes.

© 2024 Zhang J et al. *JAMA Network Open.*

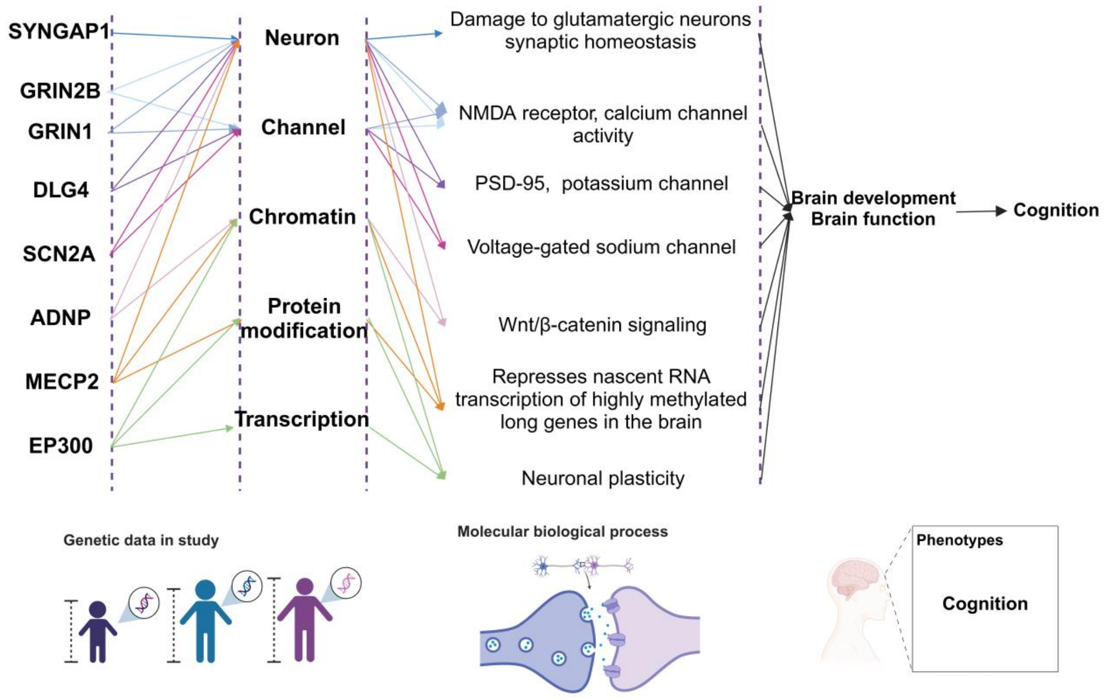


**eFigure 6. Genetic variations-brain development-phenotypic relationship network.** Genetic variations play a crucial role in shaping the intricate network that governs brain development and its phenotypic outcomes. Genes, with their diverse biological functions and enrichment in various pathways, are essential for the proper functioning of the brain. However, any dysregulation in these genes can lead to disruptions in normal brain development and functioning. This disruption often presents as cognitive impairment (CI), which is a prevalent symptom of GDD. eReferences should be included at the end of this article.

© 2024 Zhang J et al. *JAMA Network Open.*

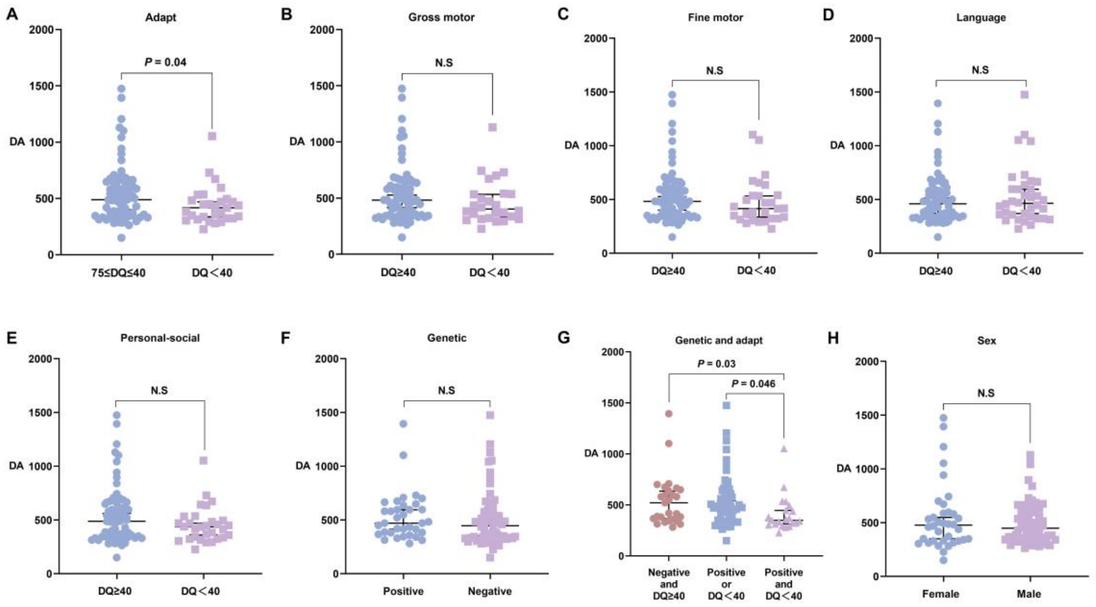


**eFigure 7. DA levels. (A-E)** Gesell adaptive area, which may reflect cognitive function, showed that patients with severe and profound cognitive function impairment (DQ < 40) had lower DA levels. While other functional regions of Gesell did not exhibit statistically significant differences in DA levels between groups, the overall trend suggested that the more severe the child's condition, the lower the DA level. **(F-G)** Analysis of the relationship between genetic factors and DA levels alone did not show statistical significance. However, when the DQ in the adaptive region was < 40 and the genetic test was positive, the DA levels of the children was lower. **(H)** There was no significant correlation between sex and DA. The upper and lower black bars denote the 95% confidence interval, while the middle black bars represent the interquartile range. N.S: No significance.

© 2024 Zhang J et al. *JAMA Network Open.*

**eReferences**


1. Ozkan ED, Creson TK, Kramár EA, et al. Reduced cognition in Syngap1 mutants is caused by isolated damage within developing forebrain excitatory neurons. *Neuron*. 2014;82(6):1317-33; doi: 10.1016/j.neuron.2014.05.015.
2. Bell S, Maussion G, Jefri M, et al. Disruption of GRIN2B Impairs Differentiation in Human Neurons. *Stem Cell Reports*. 2018;11(1):183-196; doi: 10.1016/j.stemcr.2018.05.018.
3. Santos-Gómez A, Miguez-Cabello F, Juliá-Palacios N, et al. Paradigmatic De Novo GRIN1 Variants Recapitulate Pathophysiological Mechanisms Underlying GRIN1-Related Disorder Clinical Spectrum. *Int J Mol Sci*. 2021;22(23):12656; doi: 10.3390/ijms222312656.
4. Rodríguez-Palmero A, Boerrigter MM, Gómez-Andrés D, et al. DLG4-related synaptopathy: a new rare brain disorder. *Genet Med*. 2021;23(5):888-899; doi: 10.1038/s41436-020-01075-9.
5. Meisler MH, Hill SF, Yu W. Sodium channelopathies in neurodevelopmental disorders. *Nat Rev Neurosci*. 2021;22(3):152-166; doi: 10.1038/s41583-020-00418-4.
6. Sun X, Peng X, Cao Y, et al. ADNP promotes neural differentiation by modulating Wnt/β-catenin signaling. *Nat Commun*. 2020;11(1):2984; doi: 10.1038/s41467-020-16799-0.
7. Boxer LD, Renthal W, Greben AW, et al. MeCP2 Represses the Rate of Transcriptional Initiation of Highly Methylated Long Genes. *Mol Cell*. 2020;77(2):294-309.e9; doi: 10.1016/j.molcel.2019.10.032.
8. Van Gils J, Magdinier F, Fergelot P, et al. Rubinstein-Taybi Syndrome: A Model of Epigenetic Disorder. *Genes (Basel)*. 2021;12(7):968. doi: 10.3390/genes12070968.


© 2024 Zhang J et al. *JAMA Network Open.*
