# Supplementary material for: Genetic Testing for Global Developmental Delay in Early Childhood
Source: JAMA Netw Open. 2024 Jun 5;7(6):e2415084. doi: 10.1001/jamanetworkopen.2024.15084 (PMC11154162; doi:10.1001/jamanetworkopen.2024.15084)
Supplement: Supplement 3. — Data Sharing Statement [file jamanetwopen-e2415084-s003.pdf]

Data Sharing Statement

Zhang. Genetic Testing for Global Developmental Delay in Early Childhood. *JAMA Netw Open*. Published June 05, 2024. doi:10.1001/jamanetworkopen.2024.15084

Data
Data available: No

Additional Information
Explanation for why data not available: Individual-level data are not publicly available due to ethical and legal restrictions related to the Third Affiliated Hospital of Zhengzhou University.
